# Al-, Ga-, Mg-, or Li-doped zinc oxide nanoparticles as electron transport layers for quantum dot light-emitting diodes

**DOI:** 10.1038/s41598-020-64263-2

**Published:** 2020-05-04

**Authors:** Alexei Alexandrov, Mariya Zvaigzne, Dmitri Lypenko, Igor Nabiev, Pavel Samokhvalov

**Affiliations:** 1 Laboratory of Nano-Bioengineering, National Research Nuclear University MEPhI (Moscow Engineering Physics Institute), 115409 Moscow, Russian Federation; 2 Laboratory of Electronic and Photonic Processes in Polymeric Nanostructural Materials, A.N. Frumkin Institute of Physical Chemistry and Electrochemistry of the Russian Academy of Sciences, 119071 Moscow, Russian Federation; 3 Laboratoire de Recherche en Nanosciences, LRN-EA4682, Université de Reims Champagne-Ardenne, 51100 Reims, France; 4 I.M. Sechenov First Moscow State Medical University, 119991 Moscow, Russian Federation

**Keywords:** Nanoscale materials, Nanophotonics and plasmonics, Lasers, LEDs and light sources

## Abstract

Colloidal quantum dots and other semiconductor nanocrystals are essential components of next-generation lighting and display devices. Due to their easily tunable and narrow emission band and near-unity fluorescence quantum yield, they allow cost-efficient fabrication of bright, pure-color and wide-gamut light emitting diodes (LEDs) and displays. A critical improvement in the quantum dot LED (QLED) technology was achieved when zinc oxide nanoparticles (NPs) were first introduced as an electron transport layer (ETL) material, which tremendously enhanced the device brightness and current efficiency due to the high mobility of electrons in ZnO and favorable alignment of its energy bands. During the next decade, the strategy of ZnO NP doping allowed the fabrication of QLEDs with a brightness of about 200 000 cd/m^2^ and current efficiency over 60 cd/A. On the other hand, the known ZnO doping approaches rely on a very fine tuning of the energy levels of the ZnO NP conduction band minimum; hence, selection of the appropriate dopant that would ensure the best device characteristics is often ambiguous. Here we address this problem via detailed comparison of QLEDs whose ETLs are formed by a set of ZnO NPs doped with Al, Ga, Mg, or Li. Although magnesium-doped ZnO NPs are the most common ETL material used in recently designed QLEDs, our experiments have shown that their aluminum-doped counterparts ensure better device performance in terms of brightness, current efficiency and turn-on voltage. These findings allow us to suggest ZnO NPs doped with Al as the best ETL material to be used in future QLEDs.

## Introduction

So-called quantum dots (QDs), fluorescent semiconductor nanocrystals, are of great interest today in various fields ranging from biomedicine to quantum computing^1–6^. One of the QD application areas that have recently displayed rapid progress is optoelectronics^4,7,8^, with a special emphasis on display and lighting technologies. High luminescence quantum yields, narrow emission spectra, and excellent stability of QD optical properties hold great promise for highly efficient, pure-color QD-based light-emitting diodes (QLEDs). Despite the weak electroluminescence (EL) and low external quantum efficiency (EQE) of the first devices^4^, which did not exceed 0.01%, recent QLEDs have EQEs of more than 20% with a brightness reaching 200 000 cd/m^2^^4,9^. Such a rapid and significant improvement of QLED technology was induced by the emergence of new methods for the synthesis of core/shell QDs with luminescence quantum yields as high as 100% and the optimization of the QLED structure and charge-transport materials.

First, QDs were used to form both the emitting layer and the electron-transport layer (ETL). In this device architecture, the EL spectrum had significant parasitic emission from the polymer hole-transport layer (HTL), which indicated poor exciton confinement in the QD layer^10^. Then, there was an attempt to improve the efficiency of QLEDs by using organic materials for both ETL and HTL. Although these devices displayed better characteristics^11^, their further improvement was hampered by the relatively low conductivity of the organic transport layers, which limited the injection of charge carriers, particularly electrons, into the emissive QD layer. Thus, replacement of the organic charge transport layers with inorganic materials seemed to be the solution. As it was reported^12^, the use of zinc tin oxide for an n-type charge transport layer and nickel oxide for a p-type one led to a higher current density in QLEDs, reaching 4 A/cm^2^. However, because of the considerable energy barrier between NiO and QDs, the device suffered from an insufficient hole injection rate and, hence, had an EQE lower than 0.1%.

Then, engineering of hybrid devices with an inorganic ETL and an organic HTL became the main direction of QLED evolution, which continues to the present day^4,13,14^. Although early studies reported the use of various inorganic oxides and chalcogenides as electron-transport materials^15^, ZnO has been proved to be the most favorable ETL material due to its high transparency, low work function, and high electron mobility^4,5^. Formation of ZnO ETLs for QLEDs could be done using different deposition techniques, such as the sol–gel method^16,17^, spray pyrolysis^18,19^, sputtering^20^, etc. Nevertheless, colloidal ZnO NPs have become the most widely used material for QLED ETL due to their optimal electronic structure, simple synthesis, and the possibility of using them as a wet-process-compatible conductive ink^13,21–24^. However, in the QLED structure, electron transfer from the QDs to ZnO induced by the energy difference between the conduction band minima (CBM) of these materials often leads to exciton dissociation and significant reduction of the EL efficiency. This mechanism is known as the cause of QD luminescence quenching in various QD-based systems^25–27^. In this regard, the possibility to control the CBM energy level of a ZnO-based ETL by doping can be a promising way to optimize the electronic structure and performance of the device. One of the first dopants that was used for this purpose was magnesium (Mg)^8,28,29^. Zhang *et al*. demonstrated a peak EQE of 18.2% and 18.1% for red and green QLEDs employing ZnMgO ETLs, respectively^30^. Here, a single-layer ETL consisting of Zn_0.9_Mg_0.1_O NPs, which efficiently suppressed interfacial quenching of QD luminescence, has been used. However, because it was found that the charge transfer rate and QD blinking in the multilayer structures are mostly related to the Fermi levels of metal oxide^31,32^, group-III elements, such as aluminum (Al)^33–35^ and gallium (Ga)^7^, became widely used as n-type dopants^36^ for fabrication of ZnO ETLs. For instance, the use of ZnO doped with Al (AZO) as the ETL material in solution-processed QLEDs led to a 1.8-fold enhancement of device performance compared with ZnO-based QLEDs^35^. The reason for this was effective suppression of spontaneous electron transfer at the interface of QDs and ETL, which was attributed to a decreased work function (WF) and the proper CBM alignment of the electron-transport layer and QDs, both induced by the presence of Al dopants inside the ZnO matrix. At the same time, Kim *et al*. showed that the use of solution-processed AZO with high aluminum doping levels as an ETL material in inverted red-light-emitting QLEDs promoted an increase in the maximum luminance from 6 380 to 26 700 cd/m^2^. Such a significant improvement was achieved due to the small surface roughness and reduced electrical conductivity of thin AZO films compared with ZnO^33^.

Gallium is another group-III element that is promising for QLED performance improvement. The lengths of the Ga–O and Zn–O covalent bonds are very similar to each other^37^ (1.92 and 1.97 Å, respectively), and, because the electronegativities of the Ga and Zn ions are also close, they are expected to form a Ga-doped ZnO phase without inducing crystal defects^37–39^. Red-light-emitting QLEDs with ETLs made from Ga-doped ZnO NPs exhibited a luminance as high as 44 000 cd/m^2^, which was approximately 30% higher than that of ZnO-based devices^7^. This effect was attributed to the Ga dopants, which facilitated the transfer of electrons to the adjacent QD layer and lowered the WF of the ETL.

Lithium is another prospective dopant for the ZnO ETL that can improve the QLED performance due to elimination of the interstitial zinc defects^40,41^. The results of a study on hybrid photovoltaic devices by Soultati *et al*. indicate that Li^+^ ions intercalate into the ZnO lattice and replace interstitial zinc defects, which act as trap states and give rise to a higher electron conductivity without significantly altering the WF and valence band edge^41^. A more complicated ETL composition was proposed by Kim *et al*., who used Mg and Li co-doped ZnO (MLZO) NPs to form the ETL^42^. The authors reported inverted QLEDs with an MLZO ETL that exhibited a superior device performance because of a better charge balance and fewer defect states at the QD/ETL interface compared with QLEDs with pure ZnO and Li- or Mg-doped ZnO ETLs. The external quantum efficiency of this device reached 18.4%.

As can be seen from the literature, the use of doped ZnO NPs in the ETL is a powerful approach for tuning QLED performance that allows achieving record-setting device characteristics. To the best of our knowledge, despite the large number of studies on the influence of various ZnO dopants on the QLED efficiencies, there are still no reports on comparative study of the most common doping strategies for ZnO NPs used as an ETL in the direct QLED structure. In this study, we have investigated QLEDs with ETLs consisting of ZnO NPs doped with Al, Ga, Mg, or Li, which could be helpful for selection of the best ETL materials for future development of the QLED technology.

## Experimental Section

### Materials

Cadmium oxide (99.5%, powder), 2-ethylhexanoic acid (2-EHA, 99%), 1-octadecene (ODE, technical grade, 90%), oleylamine (OLA, technical grade, 70%), hexadecylamine (technical grade, 90%), trioctylamine (98%), trioctylphisphine (TOP, technical grade, 97%), selenium powder (powder, 100 mesh, 99.5%), zinc oxide (puriss, 99–100%), thiourea (TU, ACS Reagent,>99%), triethylene glycol dimethyl ether (TEGDME, 99%), hexadecylamine (technical grade, 90%), palmitic acid (BioXtra, ≥99%) zinc acetate dihydrate (ACS reagent, ≥99.0%), magnesium acetate tetrahydrate (ACS reagent, ≥98%), aluminium chloride hexahydrate (99%), gallium nitrate hydrate (99.9%), lithium chloride (anhydrous, ACS reagent, ≥99%), tetramethylammonium chloride (TMACl, reagent grade, ≥98%), potassium hydroxide (reagent grade, 90%), dimethyl sulfoxide (DMSO, anhydrous, ≥99.9%), and monoethanolamine (MEA, ACS reagent, ≥99.0%) were purchased from Sigma-Aldrich. n-Hexadecylphosphonic acid (97%) was from PlasmaChem GmbH. Methyl acetate (extra pure, 99%) was from Acros. Hexane, n-octane, toluene, acetone, methanol, ethanol, n-butanol, and isopropyl alcohol of spectroscopy grade were purchased from the local supplier Ekos-1. All reagents were used as received, without purification.

### Synthesis of CdSe/ZnS/CdS/ZnS quantum dots

The detailed procedure for the synthesis of CdSe/ZnS/CdS/ZnS core/multishell QDs was described elsewhere^43^. Briefly, 2.3-nm CdSe core nanocrystals were obtained by means of hot injection synthesis using the cadmium hexadecylphosphonate and TOP-Se precursors in ODE at 240 °C. The obtained CdSe nanocrystals were isolated from crude solution by precipitation with methyl acetate, redispersed in toluene, filtered through a gel permeation column (Bio-Rad Bio-Beads SX-1), and treated with OLA^44^. Deposition of the ZnS/CdS/ZnS multishell was performed at 175 °C in a layer-by-layer manner in an OLA:ODE medium (1:1 v/v) using zinc and cadmium 2-ethylhexanoates in ODE and thiourea in TEGDME as precursors. Finally, the CdSe/ZnS/CdS/ZnS core/multishell QDs were isolated from the reaction mixture by precipitation with methyl acetate and then redispersed in hot n-hexane containing a pre-dissolved mixture of hexadecylamine and palmitic acid (1 mmol of each). After cooling, the QD solution was filtered through a 220-nm PTFE syringe filter, precipitated with excess of methyl acetate, centrifuged, and redissolved in n-octane to a concentration of 20 mg/ml.

### Preparation of a 0.5 M tetramethylammonium hydroxide (TMAH) solution

We found that commercially available solutions of TMAH did not allow fabrication of ZnO NPs with reproducible properties, presumably due to the batch-to-batch variation of the reagent concentration. Therefore, each synthesis of ZnO-based NPs was performed using a freshly prepared 0.5 M TMAH solution, which was obtained as follows. TMACl (4 mmol) was placed into a 25-ml beaker together with 4 ml of methanol and a small stirring bar. The beaker was sealed with Duraseal film, and the solution was stirred for 5 min until complete dissolution of TMACl was observed. KOH (4 mmol) was dissolved in 4 ml of methanol in another beaker in a similar manner and then poured into the TMACl solution under vigorous stirring. A white precipitate of KCl was formed immediately after mixing of the precursors, but the reaction was allowed to proceed for 15 min to complete the formation of TMAH. Finally, the reaction mixture was passed through a 450-nm PET syringe filter to completely remove the insoluble KCl reaction byproduct.

### Synthesis of undoped ZnO nanoparticles

The synthesis of ZnO NPs was performed according to the procedure adapted from that reported earlier^45^. Briefly, 1.5 mmol of zinc acetate dihydrate in 15 ml of DMSO was placed into a 100-ml round-bottom flask. The mixture was stirred for 5 min, until complete dissolution of the zinc salt was observed. Next, 6 ml of a 0.5 M TMAH solution was introduced into the reaction mixture during 60 s using a syringe pump, with the mixture stirred at 930 RPM. The reaction was allowed to proceed for 1 h, and the product was precipitated with 60 ml of acetone. The cloudy solution containing aggregated ZnO NPs was centrifuged at 5 000 RPM for 5 min, the supernatant was discarded, and the compact ZnO NP pellet was dissolved in a small amount of methanol. The solution was precipitated again with excess acetone and centrifuged, and the resultant ZnO NPs were dissolved by sonication in a mixture of 5 ml of ethanol, 5 ml of n-butanol, and 80 μl of MEA to a concentration of 15 mg/ml. The ZnO NP ink was stored in the dark at room temperature prior to QLED fabrication.

### Synthesis of Li_0.05_Zn_0.95_O lithium-doped (LiZnO) nanoparticles

The synthesis of LiZnO NPs was performed by the aforementioned procedure, modified according to the results published before^41^. The metal precursor solution was obtained by dissolving 0.075 mmol of LiCl in 15 ml of DMSO using sonication, subsequent addition of 1.425 mmol of zinc acetate dihydrate, and dissolution of the obtained mixture by stirring. All other synthetic operations were performed in the same manner as those for the synthesis of ZnO NPs described above.

### Synthesis of Al_0.1_Zn_0.9_O aluminium-doped (AZO) nanoparticles

AZO NPs were obtained by the method described above, modified according to the results published before^35^. Aluminum chloride hexahydrate (0.15 mmol) was sonicated in 15 ml of DMSO until a fine milky suspension was obtained. Zinc acetate dihydrate (1.35 mmol) was introduced into this suspension; after 5 s of stirring, the reaction mixture became completely transparent. The injection of 5 ml of a 0.5 M TMAH solution (6 ml/min) was started immediately after a clear solution was obtained to avoid the formation of large or aggregated AZO NPs. All other procedures were identical to those described for the synthesis of ZnO NPs.

### Synthesis of Zn_0.92_Ga_0.08_O gallium-doped (ZnGaO) nanoparticles

ZnGaO NPs with a doping level of 8%^7^ were fabricated using the procedure described for ZnO NPs except that the metal precursor solution was made of 0.12 mmol of gallium nitrate hydrate and 1.38 mmol of zinc acetate dihydrate dissolved in 15 ml of DMSO by stirring for 5 min.

### Synthesis of Zn_0.95_Mg_0.05_O magnesium-doped (ZnMgO) nanoparticles

ZnMgO NPs were fabricated according to the procedure reported before^28^. Briefly, 0.075 mmol of magnesium acetate tetrahydrate, 1.425 mmol of zinc acetate dihydrate, and 15 ml of DMSO were mixed in a 100-ml round-bottom flask by stirring at 930 RPM. Immediately after a clear solution was obtained, injection of 5 ml of 0.5 M TMAH solution (6 ml/min) was started to avoid NP overgrowth and aggregation. All other procedures were the same as those described for the synthesis of ZnO NPs.

### Fabrication of QLED devices

ITO-coated glass substrates were pretreated in an ultrasonic bath and oxygen plasma-cleaned. A PEDOT:PSS solution (Heraeus, Clevios P VP. Al 4083) was spin-coated onto these substrates at 2000 RPM for 60 s and baked at 110 °C for 10 min. The film thickness was 40 nm. The PEDOT:PSS-coated substrates were transferred into an argon-filled glove box (O_2_ < 1 ppm, H_2_O < 1 ppm). Poly-TPD (ADS254BE, American Dye Source, in chlorobenzene, 8 mg/ml) and polyvinyl carbazole (PVK, Sigma-Aldrich, average Mn = 25 000–50 000, in o-xylene, 1.5 mg/ml) were deposited by layer-by-layer spin coating at 2000 RPM for 60 s. The poly-TPD (30 nm) and PVK (5 nm) layers were baked at 110 °C for 20 min and at 140 °C for 30 min, respectively, before the deposition of the next layer. The layer of CdSe/ZnS/CdS/ZnS QDs capped with hexadecylammonium palmitate ligands was spin-cast on PVK from a 20 mg/ml solution in n-octane at 1500 RPM for 1 min and dried at a temperature of 90 °C for 10 min. The film thickness was 80 nm. Then, electron transport layers 50 nm in thickness were applied from solutions of ZnO, LiZnO, AZnO, ZnGaO, and ZnMgO NPs in an ethanol:butanol mixture (1:1 v/v, 15 mg/ml) by spin coating at a rate of 850 RPM and dried at 70 °C for 30 min. Finally, an 80-nm-thick Al cathode was deposited onto the ETL through a shadow mask by thermal evaporation under 2 × 10^−6^ mbar vacuum. The emission area of each device was 6 mm^2^.

### Instrumental methods

The absorbance and fluorescence of QDs in octane and ZnO-based NPs in methanol were measured using an Agilent Cary 60 spectrophotometer and an Agilent Cary Eclipse spectrofluorimeter. Dynamic light scattering experiments were conducted using a Malvern Zetasizer Nano ZS instrument in glass cuvettes at 25 °C using the following parameters: a solvent (DMSO) viscosity of 1.99 mPa×s, a solvent refractive index (RI) of 1.475, and a material (ZnO) RI of 1.99.

The EL spectra of QLEDs were recorded using an Avantes 2048 fiber-optic spectrofluorimeter. The voltage–current and voltage–brightness characteristics were measured with a Keithley 2601 SourceMeter 2601, a Keithley 485 picoampermeter, and a TKA-04/3 luxmeter–brightness meter. The preparation of QLED samples and measurements of their spectral and optoelectric characteristics were performed at room temperature in argon atmosphere. The thickness of the films was determined using an MII-4 interferometer.

AFM studies were performed using an Enviroscope probe microscope with a Nanoscope Bruker controller operating in the tapping mode. NSG30 silicon cantilevers, provided by TipsNano, were used, the nominal resonant frequency was 340 kHz, the hardness coefficient was 40 N/m, and the probe radius was 10 nm. Experimental data were processed using the NanoScope Analysis 1.4 software.

## Results and Discussion

### Synthesis and properties of ZnO nanoparticles

Since the seminal work by Stouwdam and Janssen^13^, the methods for fabrication of ZnO NPs intended for the use as an ETL material in photovoltaic devices and LEDs have been evolving in two general directions. The first one aims at the ZnO NP synthesis in aqueous or alcohol media, employing alkali metal hydroxides (LiOH, KOH) as the source of oxygen^23,46,47^. This method typically requires large reaction volumes, heating of the reaction mixture, and long-term or complex injection patterns to avoid undesired growth or large NPs. Another option is the synthesis of ZnO NPs in polar aprotic media, such as dimethyl sulfoxide, using alkylammonium hydroxides (TMAH) as oxygen precursors^29,45^. This method is relatively simple, requires minimum numbers of reagents and solvents, and allows reproducible synthesis of small ZnO NPs.

**Figure 1:**
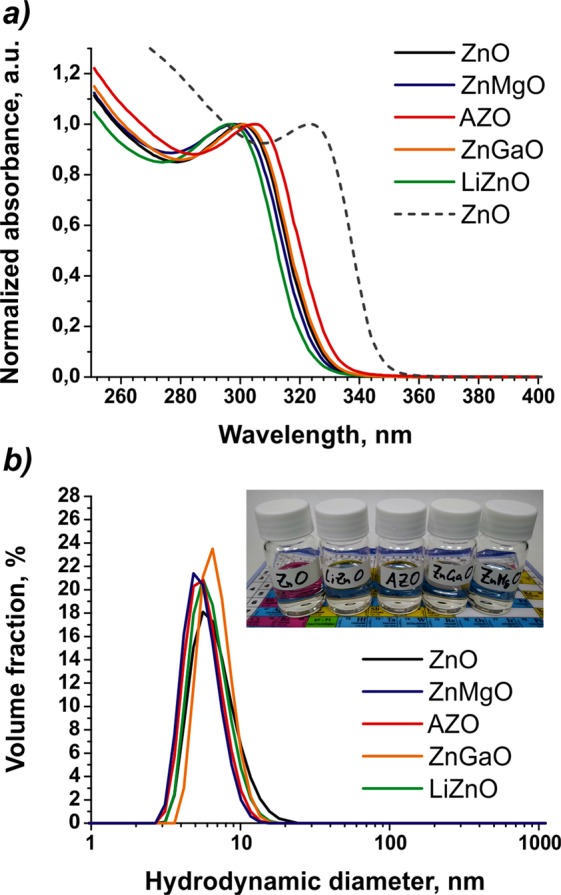
Optical absorbance spectra and size distribution by volume of the as-prepared doped and undoped ZnO nanoparticles. Optical absorbance spectra (**a**) and size distribution by volume (**b**) of ZnO (black line), ZnMgO (blue), AZO (red), ZnGaO (orange), LiZnO (green), ZnO (dashed) nanoparticles obtained with ethanol as the solvent for TMAH. The inset shows the photograph of the NP solutions used for the QLED fabrication.

In this study, the synthetic procedure reported earlier^45^ was adopted with minor modifications as the common method to obtain both doped and undoped ZnO NPs. The dopant quantities were determined according to the composition of the best-performing ETLs of QLEDs reported in the corresponding studies. Specifically, the ZnO NP doping levels were 5%, 10%, 5%, and 8% for Mg^28^, Al^35^, Li^41^, and Ga^7^, respectively. All samples displayed weak and broad fluorescence peaking at about 485 nm (Figure S1). The absorbance spectra of the obtained NPs are presented in Fig. 1a. As can be seen, the addition of dopants had a minor effect on the spectral position of the excitonic transition of the synthesized NPs. This could be due to the relatively low doping level of the NPs that are typically used as the ETL material in QLEDs. On the other hand, as it is reported in the aforementioned studies, doping affects both the CBM and VBM of ZnO-based NPs, while the band gap remains approximately at the level of pure ZnO NPs. Using Tauc’s method for direct band gap semiconductors^48^ (Figure S2), we estimated the band gaps of the obtained NPs as 3.87, 3.93, 3.82, 3.86, and 3.90 eV for ZnO, LiZnO, AZO, ZnGaO, and ZnMgO NPs, respectively. These values correlate with the ones reported in the literature. As far as we know, there is no commonly accepted sizing curve that could allow determination of the size of ZnO NPs from their optical absorbance spectra. Taking into account the absorbance data available from Meulenkamp^49^, we can estimate the size of the ZnO NPs used in this study as <2.7 nm. We used dynamic light scattering (DLS) to measure the size distribution of the obtained NP ensembles and detect aggregated NPs. The hydrodynamic diameters of the obtained NP ensembles (Fig. 1b) are characterized by a narrow distribution peaking at about 5.6 nm for ZnO, LiZnO, AZO, and ZnMgO NPs and 6.5 nm for ZnGaO NPs. These data disagree with the spectral position of the first excitonic transition in the absorbance spectra of the obtained NPs, which corresponds to a smaller NP size. However, the hydrodynamic diameter of NP is typically larger than the physical one determined by means of TEM or XRD, because the former includes the organic ligand shell and a thin layer of the surrounding medium that moves an integral part of the NP^50^. Note that the DLS results did not show the presence of aggregated NPs in any of the obtained nanoparticle samples.

In the course of optimization of the synthetic procedures, we observed that Mg- and Al-doped ZnO NPs are prone to aggregation at the early stages of reaction. Formation of a cloudy solution was accompanied by appearance of scattering onset in the absorbance spectra and distortion of the NP ensemble size distribution (see Figure S3 in the SI). We suppose that this effect was due to the high Lewis acidities of the Mg^2+^ and Al^3+^ ions, which caused premature metal acetate hydrolysis by water that was present in the reaction mixture as a component of hydrate precursors. When TMAH was injected, these hydrolyzed acetate moieties may have served as nucleation centers. These nuclei, whose amount was reduced compared to the normal reaction, started rapid growth along with the formation of new small NPs, which resulted in a highly polydisperse ensemble of small NPs, very large NPs, and their aggregates (Figure S3). Remarkably, we did not encounter this problem during the synthesis of lithium-doped ZnO NPs. The Li^+^ ion is a hard Lewis acid like Mg^2+^, but it was introduced into the reaction mixture in the form of anhydrous LiCl, which possibly eliminated the risk of early precursor hydrolysis. Nonetheless, the aforementioned early aggregation effect could be overcome by starting injection of TMAH as soon as a clear metal precursor solution is obtained, without any delay, along with inconsiderable reduction of the quantity of the injected TMAH (see the Experimental section for details).

In addition, we observed that, when ethanol was used for dispersion of the TMAH precursor during the synthesis, the size of the nanoparticles was reproducibly increased regardless of the presence of dopants (the dashed line in Fig. 1a demonstrates the effect on ZnO NPs). This could be interpreted as the solvent interference in the reaction occurring via the alcoholysis mechanism reported, e.g., in^51^. Whereas the mean size of the NPs in the ensemble was increased, the total number of nuclei at the early nucleation stage was decreased; i.e., solvent molecules had a strong effect on the reaction at the early nucleation stage. The difference between the effects caused by two structurally similar solvents may have been due to a more efficient screening of the surface of growing NPs by slightly more bulky ethanol molecules.

### Performance of QLEDs with doped and undoped ZnO ETLs

The internal efficiency of QLEDs depends on the efficiency of radiative recombination of excitons inside the QD active layer and proper balancing of the incoming electron and hole fluxes. This balancing can be achieved by using appropriate materials which have energy levels matching those of QDs, or by modulation of the electron and hole mobilities inside the device using charge carrier blocking layers^5,9,52,53^. In this study, we investigated a series of QLEDs with ETLs based on doped ZnO NPs with the structure ITO/poly(3,4-ethylenedioxythiophene):polystyrenesulfonate (PEDOT:PSS)/poly[N,N’-bis(4-butylphenyl)-N,N’-bis(phenyl)benzidine] (poly-TPD)/poly(9-vinlycarbazole) (PVK)/QDs/(Li,Mg,Al,Ga)ZnO NPs/Al. All layers were spin-coated onto a patterned ITO substrate except for the Al cathode, which was deposited through a shadow mask by thermal evaporation in vacuum (see the Experimental section). We used a thin film of CdSe/ZnS/CdS/ZnS core/multishell QDs with a photoluminescence quantum yield (PLQY) reaching nearly 100% as a light-emitting layer^43,54^. These QDs are based on relatively small CdSe cores with a diameter of 2.3 nm, with a strictly three-monolayer-thick shell, which not only ensured the small overall physical size of the whole core/shell nanocrystal (~5 nm), but also allowed achieving the highest possible confinement potentials for the excitons inside the fluorescent cores. In terms of QLED design, this means that a high electron injection barrier is formed at the interface between the QD active layer and ZnO-based ETL. This is important because most ZnO doping strategies aim at raising the CBM of the ETL to provide more efficient charge injection and suppress spontaneous charge transfer at the QD/ZnO interface. In other words, the QDs used create an extraordinary high barrier for electron injection, which may reduce the overall device efficiency but highlight the effects of ETL doping.

**Figure 2:**
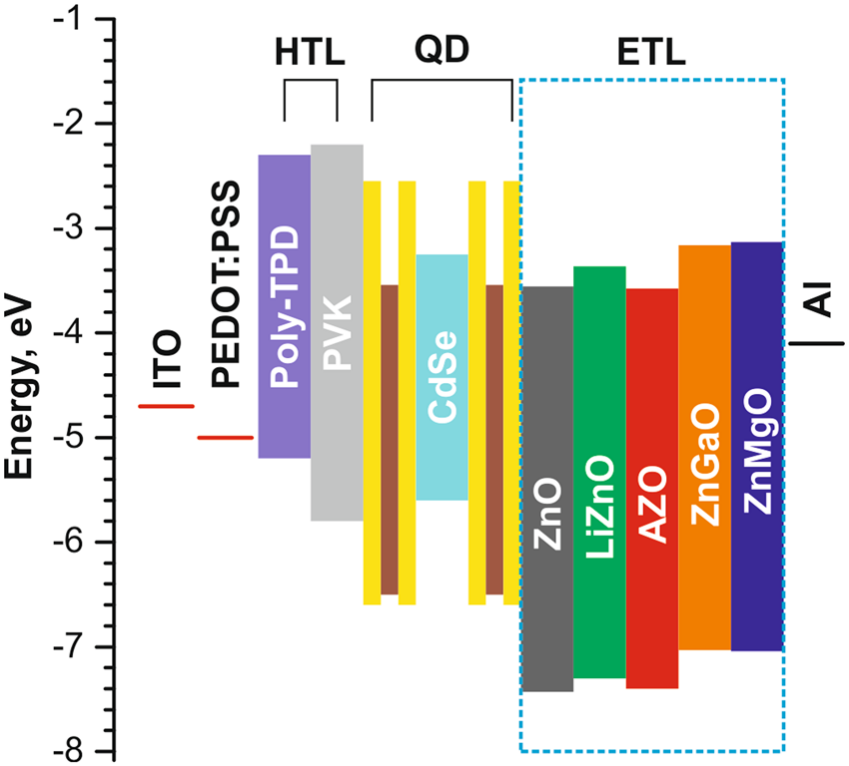
Structure and the corresponding flat-band energy levels of the QLED devices.

The schematic representation and the energy diagram of the QLED devices are shown in Fig. 2. We used a double hole transport layer (HTL) made of poly-TPD/PVK to form a stepwise change of energy levels for efficient hole injection into the QD emissive layer. To study the effects of dopants on the performance of QLEDs, thin films of undoped ZnO NPs and ZnO NPs doped with group I–II–III metals were employed as ETLs. The CBM energies of the ETL in our devices were estimated using the literature values of valence band maxima and the band gaps measured in this study. For each type of ZnO-based NPs, a series of four devices was fabricated to test the reproducibility of the QLED operation.

**Figure 3:**
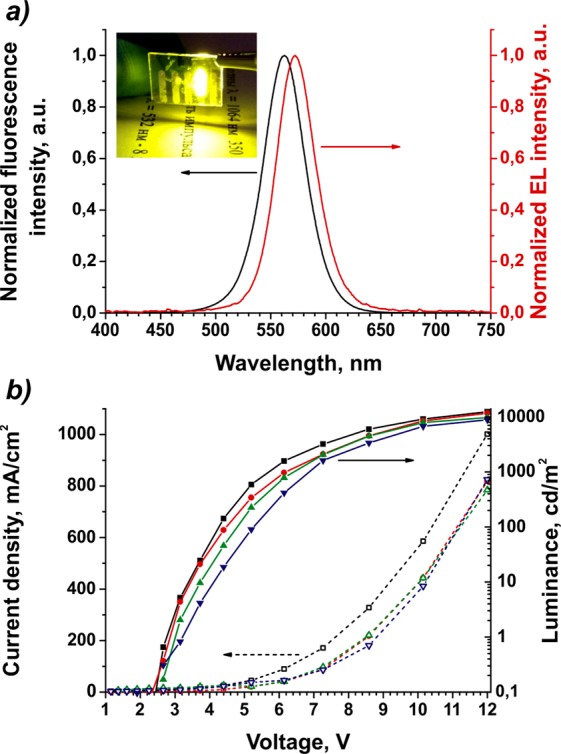
Electroluminescence of the fabricated QLEDs, fluorescence spectra of initial CdSe/ZnS/CdS/ZnS QDs, and current density and luminance versus voltage characteristics of four QLED samples employing AZO nanoparticles as an electron transport layer material. Panel a shows the electroluminescence (EL, red line) and the fluorescence (black line) spectra of the fabricated QLEDs and initial CdSe/ZnS/CdS/ZnS QDs in a solution, respectively. The inset shows a photograph of an AZO-based device driven by a 9 V source. Panel b shows current density (dashed lines, open symbols) and luminance (solid lines and symbols) versus voltage characteristics of four QLED samples employing AZO nanoparticles as an electron transport layer material.

The EL spectra of all of the fabricated devices were absolutely identical and were red-shifted relative to the initial QDs in a solution (Fig. 3a) due to fluorescence resonance energy transfer between adjacent QDs in a close-packed film^55^. Our QLEDs exhibited a pure yellow EL emission with the CIE coordinates (0.489; 0.497).

The best performance characteristics were displayed by QLEDs with AZO-based ETLs, which is unexpected, given the alignment of energy levels in this system. The maximum brightness and current efficiency of these type of QLEDs were 12 400 cd/m^2^ and 2.37 cd/A, respectively. The turn-on voltage of 2.8 V was also slightly lower for QLEDs with AZO ETLs. Figure 3b shows the current density and luminance characteristics of four AZO-based QLEDs as functions of the driving voltage. As can be seen from the figure, these parameters are highly reproducible for devices of the same series.

**Figure 4:**
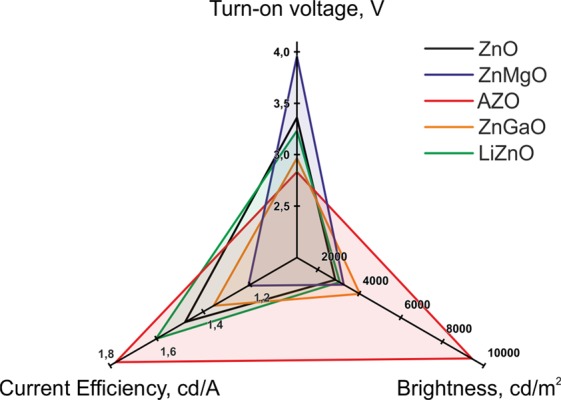
Brightness, current efficiency, and turn-on voltage of the fabricated QLEDs with different electron transport layer materials: ZnO (black line), ZnMgO (blue), AZO (red), ZnGaO (orange), LiZnO (green).

The mean values of brightness, current efficiency, and turn-on voltage characteristics of all of the fabricated QLEDs are summarized in Table 1 and Fig. 4. Compared with QLEDs with undoped ZnO NP ETLs, doping of ETLs with Li, Ga, and Mg led to a moderate improvement of the device brightness, current efficiency, and turn-on voltage (Fig. 4). These results do not correlate with the earlier studies using Ga-doped^7^ and Mg-doped^28^ ZnO ETLs, which reported a more than 50% improvement of QLED current efficiencies over the ones where undoped ZnO NPs were used, although the brightness of these devices was improved to a smaller degree. This difference may be determined by the different types of QDs used in those studies. The red-light-emitting CdSe/ZnS QDs used in^7^ had a lower CBM than CdSe/ZnS/CdS/ZnS QDs, which we used here. Therefore, the lowering of the spontaneous charge transfer efficiency in Ga-doped ZnO ETLs compared with CdSe/ZnS QDs, which was considered to be the major contributor to the enhanced QLED parameters, is less pronounced in our case because of the poorer matching of energy levels. The CBM of CuInS_2_-based QDs employed as the electroluminescent material in^28^ lies even deeper than that of CdSe QDs of any size^27^. Therefore, in the case of Mg-doped ZnO NPs, the effect of suppression of interfacial charge transfer should be less pronounced for the QLEDs based on CdSe QDs than for CuInS_2_-based devices. These results serve as a clear demonstration that enhancement of the QLED performance via chemical modification of ZnO-based ETLs is not a versatile approach and that both the size and the composition of QDs should be taken into account.

**Table 1:** Characteristics of the fabricated QLEDs.

Type of the ETL employed	Brightness, cd/m^2^	Current efficiency, cd/A	Turn-on voltage, V	Current density at 12 V, mA/cm^2^
ZnO	2 850 ± 1 526	1.48 ± 0.61	3.36 ± 0.59	423 ± 273
LiZnO	3 055 ± 1 666	1.60 ± 0.79	3.23 ± 0.23	456 ± 130
AZO	9 410 ± 2 347	1.78 ± 0.48	2.83 ± 0.20	805 ± 111
GaZnO	4 027 ± 467	1.36 ± 0.26	2.97 ± 0.06	581 ± 57
ZnMgO	3 256 ± 810	1.21 ± 0.70	3.96 ± 1.15	425 ± 209

To the best of our knowledge, this study is the first demonstration of the use of Li-doped ZnO NPs as an ETL material in QLEDs of non-inverted structure. Lithium doping of zinc oxide has been proposed for the preparation of the ETL of organic solar cells^41^. The authors reported that lithium ions intercalated into the ZnO lattice, replaced interstitial zinc defects that acted as trap states, and gave rise to a higher electron conductivity without significantly altering the WF and valence band edge of the material. This led to a considerable increase in the power conversion efficiency and, which is even more important, promoted stability of the photovoltaic devices in the ambient air. The considerable stability improvement of the Li-modified devices was attributed to the reduction of Zn^2+^ interstitial defects of ZnO upon Li doping, which prevented the adsorption of corrosive agents and boosted the stability of the Li-modified ZnO-based devices. QLEDs are intended fir operation under ambient conditions with high electrical currents; hence, their resistance to oxidation is of high importance. Although our QLEDs with a Li-doped ZnO ETL did not exhibit significant improvement of all the discussed characteristics with exception of a relatively high current efficiency, we can suggest lithium doping as a subject of future work on enhancing the atmospheric stability of QLEDs.

The maximum brightness of QLEDs with an AZO ETL exceeds the brightness of the other devices by a factor of 2.5–3, while their current efficiency is only 1.2–1.4 times higher. This could be due to an apparently higher current density at the same driving voltage in the samples with AZO ETLs than in the other QLEDs (Table 1). The high current density observed in the AZO-based devices can be related to the excess of free electrons provided by Al dopants in the ZnO NPs^35^, which is accompanied by an elevated CBM energy compared to undoped ZnO. As can be seen from Fig. 2, both ZnGaO and ZnMgO have a better CBM energy level alignment with the CdSe cores of QDs, which is expected to be a prerequisite of a high QLED performance. On the other hand, both materials have a higher valence band edge, which could lead to a less efficient hole blocking causing the formation of leakage current and generating the Joule heat. This, together with the aforementioned different energy structure of QDs used in this study, gives rise to the overall moderate increase in the efficiency of the ZnGaO- and ZnMgO-based devices.

**Figure 5:**
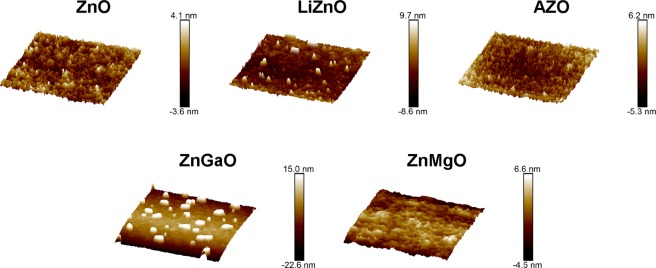
Topographic atomic force microscopy images of the top ZnO/doped ZnO nanoparticle layers of the fabricated QLEDs.

Finally, we studied the top surface layer of our QLEDs by atomic force microscopy (AFM) to correlate the performance of the fabricated devices with the homogeneity of ETL films made of doped and undoped ZnO NPs. As seen in topographic AFM images (Fig. 5), the surface of AZO-, ZnO-, LiZnO-, and ZnMgO-based devices was relatively smooth and exhibited only a minor amount of aggregated small NPs, which stuck out of the surface no farther than one nanoparticle diameter. The RMS roughness of these films was determined to be 1.167, 1.002, 2.241, and 2.309 nm for AZO-, ZnO-, LiZnO-, and ZnMgO-based devices, respectively. In contrast, Ga-doped ZnO ETLs were characterized by large agglomerates that protrude from the relatively smooth film surface with an RMS roughness of 1.059 nm farther than 30 nm. It should be noted that no such aggregates were revealed by DLS after the synthesis and purification of ZnGaO NPs, which means that these aggregates could form during the storage of the nanoparticle ink or in the course of QLED fabrication. We believe that these aggregates could hamper uniform charge transport in the studied devices, which resulted in only moderate improvement of the performance of ZnGaO-based QLEDs compared to isoelectronic AZO-based devices. In contrast, the small RMS roughness of AZO ETLs could facilitate the electrical contact with both QD emissive layer and Al-cathode, thus ensuring a high current density in these devices and their remarkable brightness compared to other devices.

To conclude, we have observed a remarkable difference in performance between the QLEDs that were fabricated following the same routine, using the same types of QDs and hole transport layers, but with different types of the ETL material based on doped and undped ZnO NPs. Moreover, all of the ZnO-based NPs were practically identical in terms of their physical size and band gap energy. On the other hand, doping resulted in a considerable difference in the energy levels of the conduction and valence bands of the fabricated ETLs. However, the devices that had ETLs based on aluminum-doped ZnO NPs exhibited better performance than QLEDs with more advantageous band alignment between the active layer and ETL, such as ZnMgO and ZnGaO-based devices. We believe that this counterintuitive effect is a result of a more efficient carrier transport in the thin AZO film, which was reported in earlier study^35^ resulting from the excess electrons supplied by the Al dopant, along with the rather low roughness of the film, which allowed a good electrical contact with both the QD active layer and the Al cathode. This finding allows us to propose that charge transport is the more important issue which should be considered in the design of QLEDs.

## Conclusions

Recent progress in the development of highly efficient QLEDs has become available due to many factors, and the use of ZnO NP–based ETLs is an outstanding improvement which has ensured high current densities and record-setting device brightness. Several ETL doping strategies have been reported recently that allow further enhancement of QLED characteristics trough optimal alignment of the energy levels of the device components, modulation of the electron transport, and reduction of undesirable charge carrier migration between the QD active layer and the ETL. However, selection of the proper ZnO NP–based ETL material is often ambiguous, because doping with different elements has been reported to lead to approximately similar device characteristics. In our experiments, we have observed that doping of the ETL of non-inverted QLEDs with aluminum ensures the best device performance in terms of brightness, current efficiency, and turn-on voltage. Surprisingly, this is not due to the best energy alignment of CBM of both QDs and AZO NPs, but rather because of the high electron conductivity and low surface roughness of this material. Therefore, we propose AZO NPs with a doping level of 10% as the best candidate to be used in the ETL of future QLEDs. On the other hand, caution should be taken in selecting the ETL material for QLEDs based on luminescent materials other than CdSe, because their energy structure could raise the efficiency of other doped ZnO NPs forming an ETL due to the difference in the band levels and surface properties of the nanocrystal film.

## Supplementary information

Supplementary Information.
