# Supplementary material for: Al-, Ga-, Mg-, or Li-doped zinc oxide nanoparticles as electron transport layers for quantum dot light-emitting diodes
Source: Sci Rep. 2020 May 4;10:7496. doi: 10.1038/s41598-020-64263-2 (PMC7198560; doi:10.1038/s41598-020-64263-2)
Supplement: Supplementary file 1 — Supplementary Information. [file 41598_2020_64263_MOESM1_ESM.pdf]

Supporting information

Al-, Ga-, Mg-, or Li-doped zinc oxide nanoparticles as electron transport layers for quantum dot light-emitting diodes

Alexei Alexandrov[1,2], Mariya Zvaigzne[1], Dmitri Lypenko[1,2], Igor Nabiev[1,3,4*] and Pavel Samokhvalov[1*]

[1] Laboratory of Nano-Bioengineering, National Research Nuclear University MEPhI (Moscow Engineering Physics Institute), 115409 Moscow, Russian Federation.

[2] Laboratory of Electronic and Photonic Processes in Polymeric Nanostructural Materials, A.N. Frumkin Institute of Physical Chemistry and Electrochemistry of the Russian Academy of Sciences, 119071 Moscow, Russian Federation.

[3] Laboratoire de Recherche en Nanosciences, LRN-EA4682, Université de Reims Champagne-Ardenne, 51100 Reims, France

[4] I.M. Sechenov First Moscow State Medical University, 119991 Moscow, Russian Federation.

* email: p.samokhvalov@gmail.com or igor.nabiev@univ-reims.fr

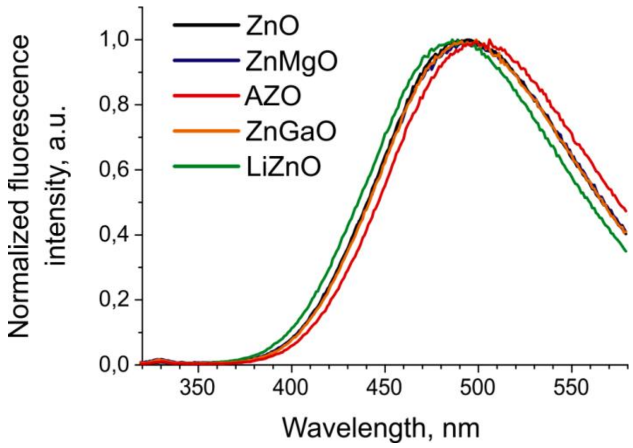

Figure S1. Fluorescence spectra of the as-prepared doped and undoped ZnO nanoparticles. Black: ZnO; blue: ZnMgO; red: AZO; orange: ZnGaO; green: LiZnO.

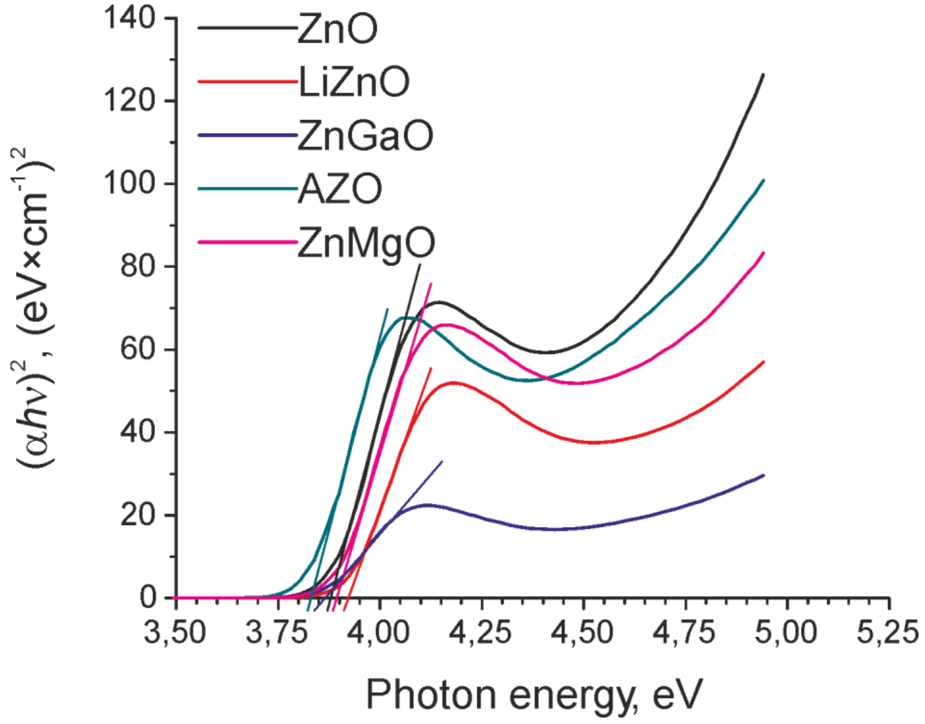

Figure S2. Tauc's plots for the obtained ZnO nanoparticle solutions.

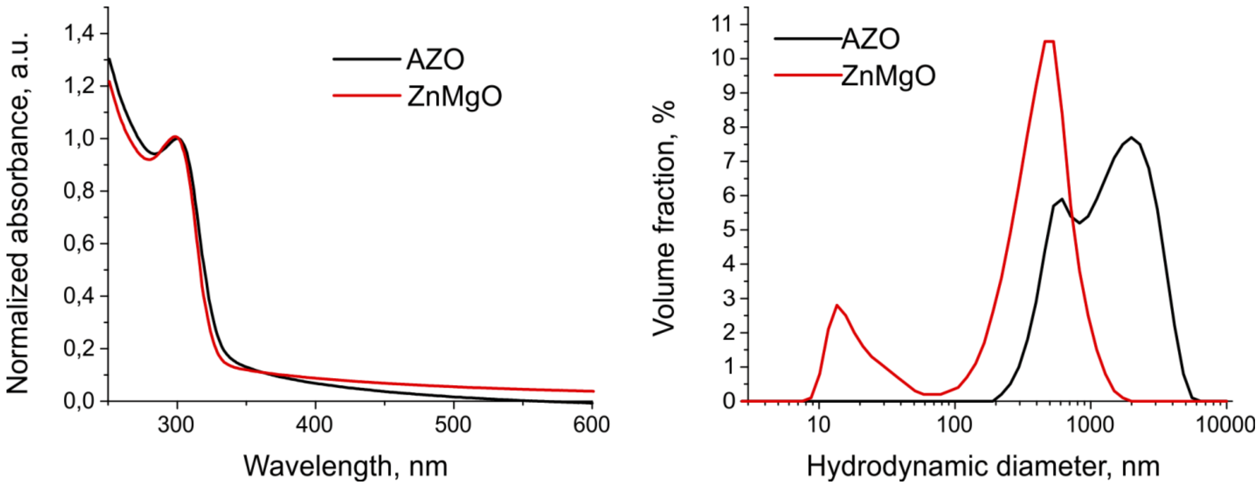

Figure S3. Signatures of *in situ* nanoparticle aggregation during the synthesis of Mg- and Al-doped ZnO nanoparticles in the absorbance spectra (left) and ensemble size distribution (right).
